# Insomnia Subtypes in Clinical Population According to the Insomnia Type Questionnaire (ITQ): A Multi‐Centre Study in Spanish Sleep Clinics

**DOI:** 10.1111/jsr.70116

**Published:** 2025-06-25

**Authors:** Francesca Canellas, Manuel de Entrambasaguas, Odile Romero, Ainhoa Álvarez, Rybel Wix, Francisco Javier Puertas, Jesús Pujol, Guillem Frontera

**Affiliations:** ^1^ Hospital Universitari Son Espases, Health Research Institute of the Balearic Islands (IdISBa), Institut Universitari D'investigacions en Ciències de la Salut (IUNICS) Universitat de les Illes Balears Palma Spain; ^2^ Hospital Clínic Universitari de València, INCLIVA Biomedical Research Institute (Valencia) Valencia Spain; ^3^ Hospital Universitari Vall D'hebron (Barcelona) Barcelona Spain; ^4^ Bioaraba Heath Research, Neuroscience, Sleep Disorders Vitoria‐Gasteiz Spain; ^5^ Hospital Universitario de la Princesa (Madrid), Instituto de Investigación Sanitaria HM Hospitales (Madrid) Madrid Spain; ^6^ Hospital Universitario de La Ribera‐FISABIO); Facultad de Medicina y CCSS, Universidad Católica de Valencia (Valencia) Valencia Spain; ^7^ ABS Balaguer, Unitat Docent Multiprofessional D'atenció Familiar i Comunitària de Lleida, Institut Català de la Salut. Barcelona Barcelona Spain; ^8^ Hospital Universitari Son Espases; Health Research Institute of the Balearic Islands (IdISBa) Palma Spain

**Keywords:** anxiety, depression, insomnia, insomnia subtypes, insomnia type questionnaire, psychopathology

## Abstract

The lack of robust subtyping for insomnia disorder (ID) led to its current classification as a uniform condition. A novel approach to subtyping ID developed a new tool, the insomnia type questionnaire (ITQ). Our research aimed to assess whether the ID subtypes identified in the general population could also be found in ID patients referred to sleep clinics in a multi‐centre study throughout Spain, and to gather insights for the management of complex ID patients. The ITQ classified ID patients into the five previously described subtypes: Type 1 = 35.1%, Type 2 = 12%, Type 3 = 46.7%, Type 4 = 5.8% and Type 5 = 0.4%. Compared to the general population, there was an overrepresentation of Types 1 and 3, consistently across all participating clinical centres. The total self‐reported sleep duration was 4.7 (SD 1.2) h, with no significant differences between subtypes. Type 1 patients had significantly higher scores in the Insomnia Severity Index, Inventory of Depressive Symptomatology self‐rated and State–Trait Anxiety Inventory. Type 3 patients were more worried about their sleep. Type 4 had the lowest depression and anxiety scores. ITQ subtyping showed that ID patients attending sleep clinics had high scores in depression, specially Type 1 patients, who probably need a differentiated therapeutic approach. The over‐representation of Type 3 patients suggests that they are more worried about their sleep than the other subtypes. These findings highlight the difficulties faced by sleep clinicians to treat complex and refractory‐toto‐treatment ID patients and those with comorbid insomnia.

## 1 Introduction

Insomnia disorder (ID) is the most prevalent sleep disorder and a significant public health problem (Morin and Jarrin 2022). ID now affects around 10% of the general adult population (Riemann et al. 2022), including 14% in Spain (De Entrambasaguas et al. 2023), where it has doubled in the last 20 years (Ohayon and Sagales 2010). In addition, almost one third of patients in a psychiatric population suffer from coexisting ID, especially those with anxiety and depression (Seow et al. 2018).

ID is considered to be the result of neurobiological vulnerability, with symptoms often triggered by life events that cause persistent physiological, cognitive and emotional hyperarousal, and is further entangled by maladaptive behaviour and dysfunctional beliefs about sleep (Riemann et al. 2022). Insomnia shows strong familial aggregation (Jarrin et al. 2017), with a reported heritability of around 0.40 in twin studies (Madrid‐Valero et al. 2021). Genome‐wide analyses have failed to link insomnia to the circuitry regulating sleep but instead have found a substantial overlap between insomnia and the circuitry involved in emotion regulation, cardiovascular and metabolic traits, restless legs and sleep duration (Jansen et al. 2019; Lane et al. 2019; Van Someren 2021). Indeed, insomnia increases the risk for psychopathology (Hertenstein et al. 2023). Cognitive‐behavioural therapy for insomnia (CBT‐I) is the recommended first‐line treatment for all adults with chronic ID, including comorbid ID (Qaseem et al. 2016; Riemann et al. 2023), but it is not effectively alike for all patients. ID may thus be viewed as a complex and heterogeneous condition where different pathways lead to the common complaint of poor sleep and related daytime impairment. Efforts to subtypify ID attempt to clarify these issues and help guide therapeutic interventions.

One initial approach was to describe primary insomnia subtypes, such as psychophysiological, paradoxical, idiopathic or inadequate sleep hygiene (ICSD‐2, American Academy of Sleep Medicine 2005), but they showed poor reliability and validity, possibly due to significant overlap with comorbid insomnia subtypes (Edinger et al. 2011) and even the original distinction between primary (non‐organic) and secondary (organic) insomnia was ultimately abandoned. Traditional top‐down subtyping based on sleep characteristics, that is, initial, middle or late insomnia, also showed poor validity because of variability both night‐to‐night (Buysse et al. 2010) and over time (Bjorøy et al. 2020).

Currently, there are two robust approaches to the phenotyping of insomnia (Nyhuis and Fernandez‐Mendoza 2023). The first one provides evidence for insomnia with short sleep duration (ISSD), which requires the recording of less than 6 h of total sleep time in polysomnography. This phenotype is related to biological vulnerability, physiological hyperarousal and an unremitting course, with patients showing a depressive‐somatic profile and an increased risk for cardiovascular, metabolic and neurocognitive morbidity (Vgontzas et al. 2013; Fernández‐Mendoza et al. 2015). This phenotype differs from the more frequent and less severe insomnia with normal sleep duration (INSD), where patients show an anxious‐ruminative profile with sleep misperception. A recent systematic review and meta‐analysis found that CBT‐I efficacy is worse in patients with ISSD than in INSD (He et al. 2023), though some studies partially or fully disagree (Rochefort et al. 2019; Crönlein et al. 2020).

The second approach used latent class analysis to take into account items predominantly unrelated to sleep but expected to be associated with insomnia, such as life history and traits of affects and personality (Blanken et al. 2019). These investigators used an online platform called the Netherlands Sleep Registry where participants from the general population voluntarily completed an ISI assessment and at least one of the 34 questionnaires (Benjamins et al. 2017). Finally, the 207‐item insomnia type questionnaire (ITQ), that included automated analysis, was constructed and validated (Suppl. to Blanken et al. 2019).

The five subtypes described by Blanken et al. 2019 depended on the extent of distress, response to pleasurable emotions and reactivity to the environment and life‐time events and could not be found with the bottom‐up subtyping of people without insomnia. Type 1 (highly distressed) was characterised by high pre‐sleep arousal, negative affect and reduced subjective happiness. Type 2 (moderately distressed, reward‐sensitive) included moderate general distress, pre‐sleep arousal, insomnia response to stress and negative affect. This subtype would be equivalent to psychophysiological insomnia. Type 3 (moderately distressed, reward‐insensitive) was characterised by overall reduced positivity. Type 4 (slightly distressed, high reactive) presented a long‐lasting insomnia response after a life event. Type 5 (slightly distressed, low reactive) showed reduced experience of pleasure and fatigue and was less reactive than Type 4, including the duration and severity of insomnia response to life events and the frequency of childhood trauma. No differences were found among insomnia subtypes in sleep onset or maintenance complaints or in total sleep time.

The distribution of these five subtypes in the surveyed Dutch general population was as follows: Type 1 = 19%, Type 2 = 31%, Type 3 = 15%, Type 4 = 20% and Type 5 = 15%. Recently, this same team found differentiating deviations in brain structural connectivity in all these subtypes, except for Type 3 (Bresser et al. 2025).

The objective of the research presented here was, first, to examine which of the insomnia subtypes identified by Blanken et al. 2019 in the general population are represented among patients with insomnia referred to sleep clinics and which subtypes do not typically reach specialist care. The second objective was to assess whether other specific clinical features appeared in patients with these ITQ subtypes. Understanding which insomnia subtypes are more likely to reach specialised sleep clinics, along with their distinguishing clinical characteristics, is essential for optimising the management ID in specialised healthcare.

## 2 Methods

### 2.1 Design

This was a multi‐centre, prospective, observational study conducted in eight sleep disorders units throughout Spain: Son Espases University Hospital (Palma, Mallorca, reference centre), University Clinical Hospital of Valencia (Valencia), Araba University Hospital (Vitoria‐Gasteiz), Vall d'Hebron University Hospital (Barcelona), La Princesa University Hospital (Madrid), La Ribera University Hospital (Alzira, Valencia), all of which belong to the public Spanish National Health System, HM Hospitales (Madrid) and Estivill Sleep Clinic (Barcelona), which are private. The subjects included were drawn from patients routinely seen at these centres. They were referred mainly by primary care and other medical specialists for diagnosis and treatment following standard clinical practice. Most of these patients had long‐standing insomnia, perhaps an unclear diagnosis or comorbidities and previous pharmacological treatments were ineffective, they refused them, or they sought for more individualised and specialised care, including CBT‐I.

Patients were assessed by a board‐certified somnologist physician in the participating sleep disorders units. Inclusion criteria were ≥ 18 years old, meeting DSM‐5/ICSD‐3 (American Psychiatric Association 2013; American Academy of Sleep Medicine 2014) diagnostic criteria for chronic ID and having an ISI > 10 at the time of assessment. This second condition was used to enhance the diagnostic criteria to include those patients with clinically significant ID. Exclusion criteria were alcohol consumption > 20 g/day in women and 30 g/day in men, substance abuse (cocaine, heroin and other drugs of abuse), and comorbid medical conditions or psychiatric disorders that fully explained insomnia. Those fulfilling inclusion criteria were informed about the objective of this research and signed an informed consent approved by the Research Ethics Committee of the Balearic Islands (CEI‐IB IB4280‐20PI) as reference centre on 9 November 2020, and then by the local Committees of all participating centres. Recruitment took place between February 2021 and February 2024.

### 2.2 Assessment Instruments

To harmonise the information collected and unify the inclusion/exclusion criteria between the different centres a structured clinical interview was developed specifically for this study. This was oriented to confirm the diagnosis of ID disorder criteria (ICSD‐3 and DSM‐5 diagnostic criteria, including notes related to differential diagnosis and exclusion criteria). In addition, it also covered ISI (Morin et al. 2011), sociodemographic descriptors, educational level (UNESCO Institute for Statistics 2012), family history of insomnia and mental illnesses, personal medical history, sleep history, sleep habits and current treatment for insomnia and other illnesses. ID comorbidities were allowed if they could not better explain the predominant presence of insomnia, following criteria as described by ICSD‐3/DSM 5.

Patients were asked about any sleep medication they were currently taking, both prescribed drugs and non‐prescribed sleep aids, including over the counter substances. This information was confirmed by checking the patient's prescriptions in the electronic medical records. The same procedure was done for treatments for other diseases.

A Spanish version of the ITQ (Blanken et al. 2019) was developed by translating this questionnaire from English to Spanish and blindly translating the Spanish version back into English until both versions matched, as suggested by Van Someren's team. We used the automated application for the calculation of the ITQ score and subtype classification, available online at https://tfblanken.shinyapps.io/itqapp/ (Suppl. to Blanken et al. 2019). Subtype calculation using the automated ITQ scoring script requires that not a single response be missing. The assignment of a patient to a specific insomnia subtype results from the highest probability, measured in %, of belonging to each of them. However, some individuals may show similar probabilities of belonging to more than one subtype, indicating uncertainty in their subtyping.

Self‐administered Spanish versions of other questionnaires were used to avoid disparities in interpretation among the participating centres. They included the Pittsburgh Sleep Quality Index (PSQI) (Buysse et al. 1989; Royuela‐Rico and Macías‐Fernández 1997); the State–Trait Anxiety Inventory (STAI) test (Buela‐Casal et al. 2011), which evaluates anxiety as a stable trait over time and as a transitory emotional state; and the Inventory of Depressive Symptomatology, self‐rated (IDS‐SR_30_) (Rush et al. 1996), which can be used both to screen for depressive symptoms and to measure their severity in the past 7 days.

The investigators ensured the privacy and data protection of this project by complying with the principles of the Declaration of Helsinki (1991) and the Spanish Organic Law 3/2018 (Boletin Oficial del Estado 2018). Study data were collected and managed using REDCap electronic data capture tools hosted at the Universitat de les Illes Balears. REDCap (Research Electronic Data Capture, Vanderbilt University, Nashville TN) is a secure, web‐based software platform designed to support data capture for research studies. The descriptive analysis included mean and standard deviation or median and quartiles for quantitative variables, and proportions for qualitative variables. The statistical significance of group differences was assessed by ANOVA and post hoc Bonferroni's test for quantitative variables, and by the Newcombe–Wilson test, based on the chi‐square test, for qualitative variables. Microsoft Excel, IBM SPSS Statistics and R software were used for the calculations.

### 2.3 Procedure

The structured interview was conducted by the researchers, all trained sleep physicians with expertise in insomnia and psychiatric comorbidities, accompanied by the patient.

Patients could complete ITQ and the other questionnaires directly on the electronic data platform, or in paper for subsequent return to the researcher, not later than 2 weeks after the initial visit. Patients could be contacted again if any item was missing.

## 3 Results

### 3.1 ITQ Distribution by Clinical Variables and ISI Score

A total of 300 patients signed the informed consent form, of whom 30 were excluded due to loss to follow‐up, one due to decease and one for developing a major depressive disorder before completing the questionnaires. Data from 268 patients included in the database were therefore analysed, although only 259 of them had a valid ITQ due to missing answers, despite the investigators efforts to fully complete the test. They included 171 women (66%) and 88 men (34%), aged 54.2 (SD 11.6) years The automated online scoring script for ITQ classified these patients into the 5 groups described in the original study, as shown in Table 1: Type 1 = 35.1%, Type 2 = 12%, Type 3 = 46.7%, Type 4 = 5.8% and Type 5 = 0.4%, as it only included one male patient, so that results by variable for this subtype are not presented. Centre membership did not influence the distribution of insomnia subtypes.

**TABLE 1:** Results of demographic variables.

	ITQ completed		Insomnia disorder subtype								*p*
Demographics	All		1		2		3		4		
Number (% subtype)	259	100	91	35.1	31	12.0	121	46.7	15	5.8	< 0.001
Women (*n*, %)	171	66.0	67	39.2	23	13.5	72	42.1	9	5.3	0.101
Men (*n*, %)	88	34.0	24	27.3	8	9.1	49	55.7	6	6.8	
Age (mean, SD)	54.2	11.6	51.1	12.1	54.9	11.4	55.4	10.6	61.5	13.3	0.012
BMI (mean, SD)	24.8	4.4	24.8	5.2	24.5	3.9	25.0	4.2	24.6	3.3	0.626
Partner status											
Married (*n*, %)	148	57.1	49	33.1	21	14.2	66	44.6	12	8.1	0.617
Divorced—separate (*n*, %)	44	17.0	19	43.2	4	9.1	20	45.5	1	2.3	
Single (*n*, %)	56	21.6	21	37.5	4	7.1	28	50.0	2	3.6	
Widower (*n*, %)	10	3.9	2	20	2	20	6	60	0	0	
Educational level											
None (*n*, %)	3	1.2	1	33.3	1	33.3	0	0	1	33.3	0.059
Basic < 8 years (*n*, %)	34	13.1	9	26.5	3	8.8	18	52.9	4	11.8	
Medium 8–12 (*n*, %)	89	34.4	43	48.3	10	11.2	32	36.0	4	4.5	
High > 12 years (*n*, %)	131	50.6	38	29.0	17	13.0	69	52.7	6	4.6	
Family history (first‐degree relatives)											
Insomnia (*n*, %)	95	36.6	38	40.0	11	11.6	43	45.3	3	3.2	0.806
Psiquiatrics (*n*, %)^a^	111	42.8	49	44.1	9	8.1	50	45.0	3	2.7	0.090
Evolution of insomnia											
Age at diagnostic of insomnia, years (median, quartiles)	41	31–53	40	30–47	43	28–56	44	31–55	56	48–68	0.007
Illness duration, years (median, quartiles)	7	1–20	8	2–20	10	1–22	6	1–19	3	2–7	0.336

^a^ Psiquiatrics: depression, anxiety, or other psychiatric illness (e.g., bipolar disorder. schizophrenia).

The most frequent subtype in both sexes was Type 3, which accounted for 42.1% of women and 55.7% of men. Type 4 patients were slightly older at 65.5 years compared to 51–55 years for the other subtypes (*p* = 0.012). There were no statistical differences across subtypes regarding body mass index, partner status, or educational level. Family history of psychiatric illness, including depression, anxiety and others such as bipolar disorder or schizophrenia, was 42.8%, more frequent than insomnia antecedents, which was 36.6%. Both familial antecedents were more frequent in Types 1 and 3 than in Types 2 and 4, although no significant statistical differences were found. Type 4 patients were diagnosed with insomnia at a significantly older age than the rest (*p* = 0.007). Duration of insomnia had a median of 7 years with a wide temporal range (p25–p75, 1–20), and tended to be shorter in Type 4, with no significant differences across subtypes. The reported current sleep duration was 4.7 (SD 1.2) h for both women and men, and there were no differences across subtypes.

ISI mean total score at the inclusion visit for 259 patients was 19.39 (SD 4.82); men: 19.26 (SD 4.50); women: 19.46 (SD 5.00), with no statistical differences across insomnia subtypes (Type 1: 20.24 SD 4.78, Type 2: 19.61 SD 5.49, Type 3 19.03 SD 4.70, Type 4: 17.40 SD 4.01, *p* = 0.149). ISI mean total score extracted from ITQ subsequently completed by the patients was 17.56 (SD 4.96). Type 1 patients had a score 19.38 (SD 4.80) significantly higher than those of Types 2–4 (*p* < 0.001), which scored around 16.00 (see Table 2). There were no statistical differences among subtypes regarding night sleep complaints but Type 3 patients were more worried about their sleep (*p* = 0.002).

**TABLE 2:** Sleep and medical conditions.

*N*			Insomnia disorder type								*p*
	Global		1		2		3		4		
	259		91		31		121		15		
	Mean	SD	Mean	SD	Mean	SD	Mean	SD	Mean	SD	
Insomnia severity index (mean, SD)											
Total ISI score	17.56	4.96	19.38	4.80	16.16	5.63	16.76	4.64	16.00	4.29	< 0.001
Difficulty initiating sleep.	1.97	1.30	2.01	1.30	1.55	1.39	2.04	1.37	2.00	1.25	0.386
Difficulty maintaining sleep	2.74	1.34	2.84	0.93	2.6	1.14	2.71	1.04	2.60	0.91	0.853
Early morning awakening.	2.38	1.00	2.52	1.19	2.32	1.30	2.34	1.31	2.13	1.55	0.637
Dissatisfied with sleep.	3.02	1.28	3.03	1.14	3.00	1.18	3.02	1.05	2.93	0.80	0.921
Interference with daily functioning.	2.79	1.08	2.64	1.22	2.68	1.19	2.96	0.99	2.67	0.98	0.169
Noticeable impaired quality of life.	1.75	1.11	1.74	1.25	1.74	1.29	1.81	1.17	1.47	1.30	0.807
Worried about sleep.	2.90	1.22	2.80	0.91	2.52	1.18	3.13	0.82	2.60	0.99	0.002
Sleep duration											
Hours	4.7	1.2	4.7	1.2	4.6	1.2	4.7	1.2	5.0	1.5	0.579

	*n*	%	*n*	%	*n*	%	*n*	%	*n*	%	
Comorbid diagnosed sleep disorders (*n*, %)											
7A4Z sleep breath dis	43	17.1	15	17.2	7	22.6	18	15.3	3	20.0	
7A60 circadian sleep dis											
Delay	8	3.2	3	3.4	0	0.0	4	3.4	1	7.1	
Advance	5	2.0	1	1.1	0	0.0	3	2.5	1	6.7	
7A87 Mov relat sleep dis	21	8.5	9	10.3	3	10.0	7	6.1	2	13.3	
Comorbid conditions that may cause sleep/wake complaints (*n*, %)											
Chronic pain	110	43.3	48	53.9	10	33.3	45	37.8	6	40.0	
Gastric or oesophageal symptoms	63	25.0	25	28.4	8	26.7	27	22.9	3	20.0	
Nocturia	95	37.1	42	46.7	9	29.0	38	31.9	6	40.0	
Comorbid medical disorders ICE‐11 (*n*, %)											
1 infections	2	0.8	2	2.2	0		0		0		
2 Neoplasms	8	3.1	3	3.3	2	6.5	3	2.5	0		
3 Blood	2	0.8	0		0		1	0.8	0		
4 Immunity	2	0.8	0		1	3.2	1	0.8	0		
5 Endocrine and metabolic	31	12.0	13	14.3	2	6.4	15	12.4	1	6.7	
8 Nervous system	9	3.5	4	4.4			4	3.4	1	6.7	
9 Visual	4	1.5	1	1.1	1	3.2	2	1.7			
10 Ear	5	1.9	1	1.1			4	3.3			
11 Circulatory system	27	10.8	8	8.8	5	16.1	11	9.1	3	20.0	
12 Respiratory system	12	4.6	5	5.5			7	5.8			
13 Digestive	13	5.0	5	5.5			8	6.6			
14 Skin											
15 Musculoskeletal	16	6.2	6	6.6	1	3.2	7	5.8	2	13.3	
16 Genitourinary	2	0.8					2	1.6			
Comorbid psychiatric disorders ICE‐11 (*n*, %)											
Anxiety	110	48.2	54	60.0	12	38.7	41	34.2	2	13.3	< 0.001
Depression	83	32.9	46	51.1	7	22.6	29	25.2	1	6.7	< 0.001
Other	7	2.7	5	5.5			2	1.7			

Regarding psychiatric comorbidities, 48.2% of patients had been diagnosed with anxiety disorders and 32.9% with depression disorders, with significant differences between subtypes (*p* < 0.001), as Type 1 patients included 60% anxiety and 51.1% depression, while Type 4 included 13.3% anxiety and 6.7% depression.

In terms of pharmacological treatment at the inclusion visit, 188 patients (72.6%) reported taking one or more (up to 4) hypnotic drugs, while 71 (27.4%) did not use any. There were no significant differences between women and men in drug use, nor among insomnia subtypes in drug class, considering benzodiazepines, antidepressants and melatonin. In relation to benzodiazepines, 14 patients (5.4%) took two drugs concurrently. Types 1 and 3 patients, who comprised 86% of the sample, were the only subtypes taking more than two hypnotic drugs and using Z‐drugs (Type 1: 17.6% and Type 3: 14.9%) and antipsychotics (Type 1: 6.6% and Type 3: 14.9%) (see Table 3).

**TABLE 3:** Treatments.

*N*	Global	Type				
		1	2	3	4	*p*
	259	91	31	121	15	
BZD = 1	111	38	10	45	3	0.145
	42.9%	41.8%	32.3%	37.2%	20.0%	
BZD > 1	14	0	6	3	5	
	5.4%	0.0%	5.5%	5.0%	20.0%	
Z	34	16	0	18	0	0.061
	13.1%	17.6%	0.0%	14.9%	0.0%	
ATD	55	21	5	27	2	0.812
	21.2%	23.1%	16.1%	22.3%	13.3%	
MEL	37	13	3	19	2	0.923
	14.3%	14.3%	9.7%	15.7%	13.3%	
DORA	1	0	0	1	0	0.887
	0.4%	0.0%	0.0%	0.8%	0.0%	
GABA_A	9	3	0	5	1	0.778
	3.5%	3.3%	0.0%	4.1%	6.7%	
HERB	6	2	0	4	0	0.801
	2.3%	2.2%	0.0%	3.3%	0.0%	
APS	7	6	0	1	0	0.086
	2.7%	6.6%	0.0%	0.8%	0.0%	
AntiH1	2	2	0	0	0	0.236
	0.8%	2.2%	0.0%	0.0%	0.0%	
Number of treatments						
0	71	18	15	32	6	0.037
	27.4%	19.8%	48.4%	26.4%	40.0%	
1	124	47	15	55	7	
	47.9%	51.6%	45.2%	45.5%	46.7%	
2	54	20	1	30	2	
	20.8%	22.0%	6.5%	24.8%	13.3%	
3	10	6	0	4	0	
	3.9%	6.6%	0.0%	3.3%	0.0%	
4	1	0	0	1	0	
	0.4%	0.0%	0.0%	0.8%	0.0%	

Abbreviations: AntiH1, antihistaminic drugs; APS, antipsychotic agents; ATD, antidepressants; BZD, benzodiazepines; DORA, dual receptor orexin antagonist; GABA_A, modulator of the gamma aminobutyric acid A; HERB, herbal remedies; MEL, melatonin; Z, zolpidem/zopiclone.

### 3.2 ITQ Distribution by Questionnaires Scores

#### 3.2.1 PSQI

PSQI mean total score was 13.77 (SD 3.36) and alike for both sexes; men: 13.42 (SD 3.81), women: 13.96 (SD 3.10). However, Type 1 patients had a significantly higher total score than the rest, 14.73 (SD 2.90, *p* = 0.002), indicating a worse globally perceived sleep quality than the other subtypes (See Table 4). Regarding PSQI components, Type 1 patients also referred worse sleep disturbance (*p* < 0.001) and daytime dysfunction (*p* < 0.001) due to sleepiness.

**TABLE 4:** Pittsburg Sleep Quality Index (PSQI).

PSQI components	Global		Insomnia disorder subtype								*p*
			1		2		3		4		
	Mean	SD	Mean	SD	Mean	sd	Mean	SD	Mean	SD	
Overall sleep quality	2.06	0.801	2.12	0.79	2.07	0.69	2.03	0.87	1.87	0.52	0.480
Sleep Latency	2.05	0.851	2.18	0.80	2.13	0.90	1.91	0.89	2.33	0.62	0.070
Sleep duration	2.23	0.896	2.24	0.85	2.40	0.81	2.23	0.89	1.93	1.28	0.800
Sleep efficiency	2.09	1.109	2.12	1.11	2.27	0.94	2.08	1.12	1.60	1.30	0.433
Sleep disturbance	1.5	0.6	1.75	0.64	1.50	0.63	1.34	0.51	1.33	0.48	< 0.001
Sleep medication use	2.18	1.255	2.33	1.14	2.07	1.34	2.11	1.31	1.93	1.39	0.539
Daytime dysfunction due to sleepiness	1.45	1.015	1.84	0.95	1.13	1.11	1.32	0.97	0.73	0.80	< 0.001
PSQI global score	13.77	3.36	14.73	2.90	13.77	3.67	13.30	3.53	11.87	2.62	0.002

#### 3.2.2 STAI Test

**FIGURE 1:**
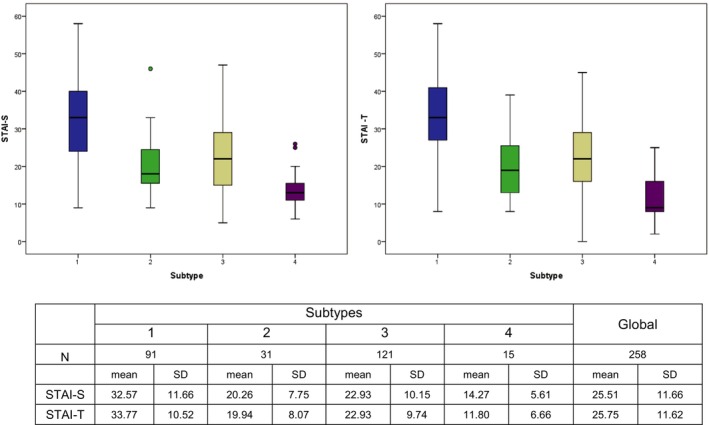
State–Trait Anxiety Inventory test (STAI).

STAI‐Trait mean total score was 25.75 (SD 11.62) alike for both sexes; men: 25.8 (SD 12.43), women: 25.99 (SD 11.20). Type 1 score was 33.77 (SD 10.52), significantly higher than that of all other subtypes (*p* < 0.0001) and Type 4 had the lowest score (*p* < 0.001) (see Figure 1). STAI‐State mean total score was 25.51 (SD 11.62) and alike for both sexes; men: 25.22 (SD 12.84), women: 25.58 (SD 11. 04). Type 1 score was 32.57 (SD 11.66), significantly higher than that of all other subtypes (*p* < 0.0001). Type 4 score was the lowest (*p* < 0.001).

#### 3.2.3 IDS‐SR_30_

**FIGURE 2:**
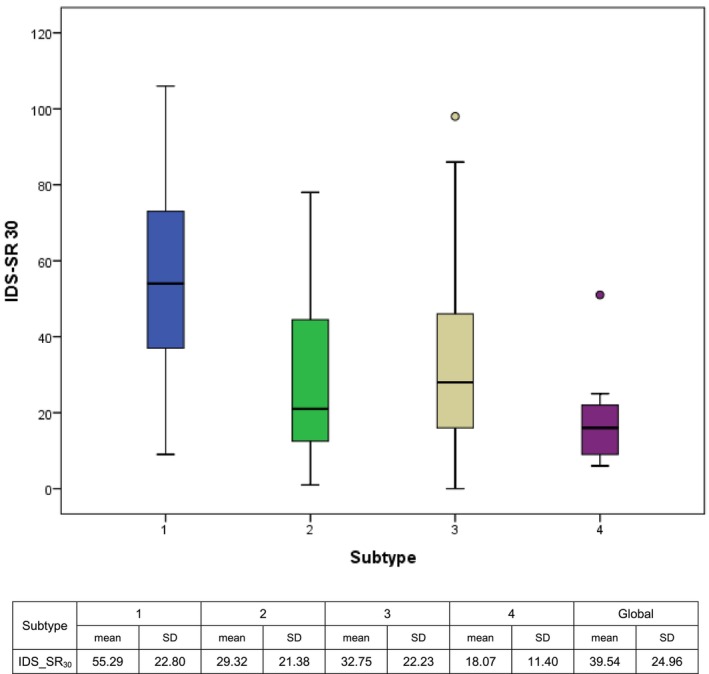
Inventory of depressive symptomatology self‐rated (IDS‐SR_30_).

IDS‐SR_30_ mean total score was 39.44 (SD 24.96) alike for both sexes (*p* = 0.185); men: 37.54 (SD 27.37), women: 40.43 (SD 23.63). Type 1 score was 55.29 (SD 22.80), significantly higher than that of all other subtypes (*p* < 0.001) and Type 4 score was the lowest (*p* < 0.002) (see Figure 2).

### 3.3 ‘Pure’ and ‘Mixed’ Subtypes

ITQ analysis showed that most patients had a clear predominant subtype. However, in some of them, there was a similarity between several subtypes, which suggested a doubtful certainty in belonging to a particular one. Thus, we considered ‘pure’ those patients in which the probability of belonging to a given subtype was twice the probability of belonging to another one, and the certainty was greater than 70%. Patients with a lower certainty were considered ‘mixed’, according to these criteria of our own.

**FIGURE 3:**
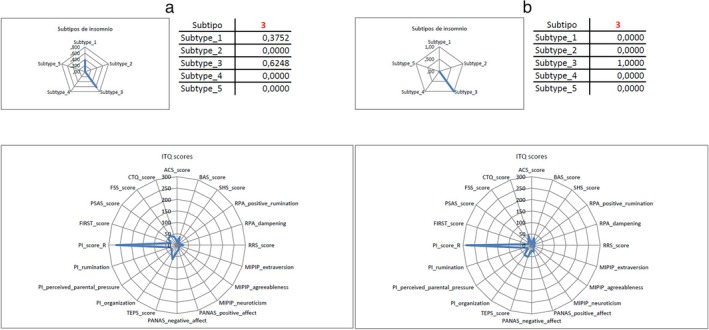
Two exemples of ‘pure’ and ‘mixed’ subtypes of insomnia. (a) pure subtype 3 of insomnia. (b) mixed subtype of insomnia.

Following the above definitions, 226 of the 259 patients (87.2%) could be considered ‘pure’ and 33 (12.8%) ‘mixed’. We searched for patterns of combination of different subtypes in a given patient but failed to find them. We also checked for differences in age and insomnia duration between ‘pure’ and ‘mixed’ subtypes and in the questionnaires scores, but no significant differences appeared. For this reason, we used the results of the whole population for the analysis. Figure 3 shows two examples of subtype calculations and results (for more information, see all results concerning ‘mixed’ and ‘pure’ subtypes in Tables S1 and S2 in supplementary data).

## 4 Discussion

Current classifications of mental, sleep and medical disorders (DSM‐5, ICSD‐3 and ICD‐11) have abandoned the distinction between primary and secondary insomnia and consider ID as an independent sleep disorder despite potential comorbid medical and/or psychiatric conditions that may in fact be dominant clinical features in some patients, a compromise that reflects the difficulty to define robust phenotypes in insomnia. However, this ‘one size fits all’ category is critically seen by sleep researchers (Riemann et al. 2022), who consider that ID cannot be regarded as a uniform condition, but as a heterogeneous disorder with different phenotypes (Van Someren 2021; Nyhuis and Fernandez‐Mendoza 2023). Identifying different insomnia subtypes can help to further develop precision medicine in insomnia and promote tailored therapeutic interventions.

The present Spanish multi‐centre study explored the frequency and distribution of ITQ types in a set of 300 patients (259 with a valid ITQ) meeting diagnostic criteria for chronic ID. The age and sex distribution of these patients was like the subjects included in the original study, and the algorithm provided by Blanken et al. allowed their classification into the same five subtypes. However, their distribution greatly differed from the original study (current vs. original): Type 1 = 35% vs. 19%, Type 2 = 12% vs. 31%, Type 3 = 47% vs. 15%, Type 4 = 6% vs. 20%; Type 5 = nearly 0% vs. 15%.

Following our criteria to describe ‘pure’ and ‘mixed’ types, only 15% of patients had a combination of characteristics of two or more subtypes. Although this low figure of ‘mixed’ subtype patients may account for the lack of findings in the analysis of this subset, it supports that ITQ is generally good at discriminating, that these subtypes are not tight, and some patients may show transitional features between them.

As compared to subjects with insomnia extracted from the general population, ID patients attended in sleep disorders clinics were more likely to be Type 1 (highly distressed) or Type 3 (moderately distressed, reward insensitive). Type 2 (moderately distressed, reward sensitive), which would correspond to psychophysiological insomnia, and especially the slightly distressed Types 4 and 5 were underrepresented in our sample, as they only accounted for 18%. Type 4 was related to childhood trauma, but in our small sample, these patients were the oldest and had the shortest duration of insomnia.

The different representation of insomnia subtypes between general and clinical population was already expected by the original authors. These differences were apparent in two elements. First, the subtype presentation. The referral of ID patients to sleep units seems related to higher levels of distress and poorer response to treatment, which describe the more frequent subtypes in our sample. Family history of insomnia (36.6%) and psychiatric illness (42.8%) were frequent, especially in Types 1 and 3 patients, although they did not reach statistical significance. This finding agrees with a recent study demonstrating the existence of genetic association between insomnia and psychiatric traits (Broberg et al. 2024). Second, the active use of sleep medication. Although Blanken et al. did not provide the % of subjects with insomnia treated with sleep medication, another publication by the same group using the same database (Netherlands Sleep Registry) reported 11.1% (19 out of 171) (Rösler et al. 2024), while in our series it was 72.6% (188 out of 259). Furthermore, patients belonging to Types 1 and 3, who represented 86% of our sample, were on combination pharmacological therapy significantly more than the other subtypes, indicating they were more reliant on medication for sleep, which was also ineffective, as they still met diagnostic criteria for clinically relevant ID. Indeed, Blanken et al. described that Type 3 patients responded worse to incidental use of any benzodiazepine than the other subtypes, while Type 2 patients, who were underrepresented in our sample, responded strongly to CBT‐I. Finally, the patients studied had been suffering from ID for a median of 7 years before being attended in a sleep clinic, which supports the claim of under‐diagnosis and insufficient health care for chronic ID (Grandner and Chakravorty 2017; Benca et al. 2023).

In addition, ISI scores were higher in our sample than in Blanken et al.'s subjects, specially in Type 1 (19.32 vs. 17.8). Both studies agree that Type 1 patients had an ISI total score significantly higher than the other subtypes when they completed ITQ. However, in our study, no differences on scores were found among subtypes in the ISI performed during the initial interview, whose scores were higher in all subtypes. It could be argued that patients, but less so for Type 1, improved in the 2 weeks allowed between the initial interview and ITQ completion, perhaps due to having started treatment, including CBT‐I. However, this discrepancy might also be due to Type 1 patients perhaps downplaying the severity of insomnia or daytime impact to the sleep physician.

PSQI scores also showed that Type 1 patients perceived worse sleep disturbance and daytime function than the other subtypes. Subjective sleep duration was worse in our sample than in Blanken et al.'s subjects. The results of both studies point to the relevance of the patient's own perception of poor sleep in insomnia.

Furthermore, STAI‐Trait and STAI‐State results confirmed that the highly distressed Type 1 patients, both men and women, were the most anxious. In addition, STAI‐Trait and State results confirmed the distinction made in the original study between the moderately distressed Types 2 and 3, and the slightly distressed Types 4 and 5 despite the low number of the latter attending the sleep clinics (Figure 1).

One of the most relevant results was the high level of depression found in all patients. The average score of IDS‐SR_30_ for all subtypes corresponded to a moderately‐severe depression (39.44 SD 24.96), with no statistical differences regarding sex. Moreover, Type 1 patients had a significantly higher score than all the other subtypes, corresponding to a severe depression (55.29 SD 22.8).

These patients consulted for poor sleep, and having a current psychiatric disorder that could explain insomnia was an exclusion criterion, however, the finding of elevated depression scores in IDS‐SR_30_ suggests that mood was not deeply explored. It also points to the intricate relationship between insomnia and depression, and the blurred borderline between ‘comorbid’ and ‘morbid’ insomnia (Riemann et al. 2022; Palagini et al. 2022). Highly distressed Type 1 patients could therefore have an upcoming depression heralded by insomnia, an established depression where poor sleep did not receive due attention, leading to a consultation with sleep specialists, or developed depressive symptoms following the existing sleep disorder. In any case, the high prevalence of Type 1 patients in our sleep clinics has four main implications. (1) The importance of early detection and treatment of insomnia to prevent depression (Cuijpers et al. 2012; Riemann et al. 2020). Insomnia needs to be routinely screened and initially treated by primary care physicians following a stepped‐care model, to promote early individualised care and prevent the development of comorbidities (Rosenberg et al. 2023; Baglioni et al. 2023). Meeting these needs requires the provision of appropriate clinical guidelines to primary care professionals. (2) Screening for current comorbid depression in patients with ID is crucial, so that assessment should not focus exclusively on insomnia symptoms. A useful tool providing a valid screening for depression is the PHQ‐9 (Kroenke et al. 2001), a 9‐item self‐administered test. (3) A differentiated therapeutic approach is needed in Type 1 patients, including psychotherapy for both insomnia and depression and the use of antidepressant drugs at antidepressant rather than just sedative doses, or the referral of these patients to mental healthcare. (4) There is a need for greater sensitivity to patients' insomnia complaints in mental healthcare, providing specific interventions, for example, CBT‐I, while excluding sleep or medical comorbidities.

Moderately distressed, reward‐insensitive Type 3 patients were the most common, accounting for nearly half of our sample. The percentage of men (55.7%) was higher than in other subtypes, as in the original study, which is intriguing. They shared with Type 1 patients a frequent family history of insomnia and psychiatric illness and the use of a greater amount and variety of medication for sleep but differed in that they had fewer comorbid psychiatric disorders. According to ISI, Type 3 patients were more worried about their sleep than the other subtypes, which could reflect their distress and difficulty in finding relief in daytime rewards. In our sample, only Types 3 and 1 patients took more than two hypnotic drugs, including Z and antipsychotics drugs. This difference in pharmacological therapy was not due to nighttime symptoms or subjective sleep duration, which were similar across subtypes. Type 3 patients had similar depression levels to Types 2 and 4 but were more anxious than Type 4 patients. Interestingly, Type 3 was the only insomnia subtype unrelated to specific structural brain connectivity (Bresser et al. 2025). It could be argued that Type 3 patients represent a more heterogeneous group of patients lacking this specific deviation, as they would exhibit a more generalised involvement of brain areas. The large representation of Type 3 patients in our sample could be related to a greater worry about sleep than the other subtypes, being more reliant on medication for sleep that was ultimately unsuccessful and lacking obvious psychiatric disorders, especially depression. Type 3 patients thus exhibited mixed characteristics, but ITQ clearly differentiated them from the other subtypes.

ID is under‐diagnosed and mis‐treated (Rosenberg et al. 2023), clinical pathways for its management in primary care are underdeveloped, including the implementation of the stepped‐care model for CBT‐I (Baglioni et al. 2023), and multidisciplinary sleep units are in short supply. Furthermore, many of the treatments proposed for ID are based on general population or primary care studies, while their lack of efficacy often results in referral to sleep units, where investigations into the features of these patients are scarce.

Our study shows that ID patients treated in sleep disorders clinics are more complex and severe than in the general population, and highlights the difficulties faced by somnologists to differentiate between ID and insomnia comorbid with psychopathology, especially depression, supporting the need to improve insomnia nosology to correctly classify patients and guide treatment (Nyhuis and Fernandez‐Mendoza 2023). In addition, it highlights the sometimes unclear boundaries between comorbid ID and insomnia as a core symptom of another sleep, medical, or mental disorder. Lack of operational definitions to distinguish between the two leaves the decision to clinical judgement. Diagnosis is an ongoing process that sometimes is not definite at the first visit and requires follow‐up, including response to different treatments, emergence of guiding symptoms and sometimes performing additional diagnostic tests. In our case, ITQ was completed early on during clinical care, which raises the question of when patients should be subtyped, as subtyping itself could help guide their treatment.

This study has some limitations. We did not perform polysomnography to all the included patients. Currently, polysomnography is only recommended to evaluate other suspected sleep disorders and in the case of treatment‐resistant insomnia (Riemann et al. 2023), but it has been argued that it should also be performed in patients with severe insomnia complaints (Fraser et al. 2023). Further research may show whether polysomnography can find an overlap between patients with severe ITQ subtypes and poor response to treatment and the ISSD phenotype (Vgontzas et al. 2013; Fernandez‐Mendoza 2017). In addition, an overlap may also be found with other undiagnosed sleep disorders. We also did not study the response to CBT‐I across subtypes or develop more specific interventions for them. With 207 items, ITQ takes some time to complete and cannot be evaluated when a single item is missing. The small number of Types 4 and 5 subjects limited the statistical analysis.

Our sample comprised patients with long‐standing insomnia and unsatisfactory response to prescribed pharmacological treatment. Under‐representation of slightly distressed subtypes could be due to them either not being diagnosed or being successfully managed in primary care. Patients with depression and anxiety symptoms are more likely to be referred to an insomnia clinic than others (Mahendran et al. 2007). Existing comorbidities, the insistence of patients to be referred, and the sensitivity of the professionals in primary care or other specialties to insomnia probably matter too.

In conclusion, we hope that our study, which to our knowledge is the first to explore ITQ in a clinical population carefully assessed by somnologists in a multicentre collaborative design, will provide some insight into the management of patients with treatment‐refractory complex ID, contribute to a better understanding of insomnia subtypes, and improve stepped‐care in patients with ID.

## Author Contributions

**Francesca Canellas:** conceptualization, investigation, funding acquisition, writing – original draft, methodology, validation, visualization, writing – review and editing, formal analysis, project administration, data curation, supervision, resources. **Manuel de Entrambasaguas:** conceptualization, investigation, writing – original draft, methodology, writing – review and editing, data curation. **Odile Romero:** conceptualization, investigation, writing – review and editing, methodology, data curation. **Ainhoa Álvarez:** conceptualization, investigation, writing – review and editing, methodology. **Rybel Wix:** conceptualization, investigation, writing – review and editing, methodology. **Francisco Javier Puertas:** investigation, writing – review and editing. **Jesús Pujol:** conceptualization, methodology, writing – review and editing. **Guillem Frontera:** conceptualization, writing – original draft, writing – review and editing, methodology, software, formal analysis, data curation.

## Conflicts of Interest

The authors declare no conflicts of interest.

## Supporting information


**Table S1.** ITQ values of ‘non‐pure’ patients (difference between the two highest certainty values, less than 50%).

**Table S2.** ITQ values of ‘pure’ patients (difference between the two highest certainty values, higher than 50%).

## Data Availability

The data that support the findings of this study are available on request from the corresponding author. The data are not publicly available due to privacy or ethical restrictions.
