# Supplementary material for: Insomnia Subtypes in Clinical Population According to the Insomnia Type Questionnaire (ITQ): A Multi‐Centre Study in Spanish Sleep Clinics
Source: J Sleep Res. 2025 Jun 25;35(1):e70116. doi: 10.1111/jsr.70116 (PMC12856099; doi:10.1111/jsr.70116)
Supplement: Supplementary file 1 — Table S1. ITQ values of ‘non‐pure’ patients (difference between the two highest certainty values, less than 50%). [file JSR-35-e70116-s002.docx]

Suppl1 table. ITQ values of “non-pure” patients (difference between the two highest certainty values, less than 50%)

| Subtype | Subtype_1 | Subtype_2 | Subtype_3 | Subtype_4 | Subtype_5 | certainty |
| --- | --- | --- | --- | --- | --- | --- |
| 2 | 0,48337 | 0,48882 | 0,02781 | 0,00000 | 0,00000 | 0,48882 |
| 3 | 0,35499 | 0,01138 | 0,37110 | 0,26253 | 0,00000 | 0,37110 |
| 1 | 0,50065 | 0,02576 | 0,47359 | 0,00000 | 0,00000 | 0,50065 |
| 1 | 0,48573 | 0,00000 | 0,06887 | 0,44540 | 0,00000 | 0,48573 |
| 1 | 0,47870 | 0,09264 | 0,42866 | 0,00000 | 0,00000 | 0,47870 |
| 3 | 0,43507 | 0,07838 | 0,48656 | 0,00000 | 0,00000 | 0,48656 |
| 3 | 0,00031 | 0,00199 | 0,53682 | 0,46036 | 0,00053 | 0,53682 |
| 1 | 0,51006 | 0,43036 | 0,00014 | 0,05944 | 0,00000 | 0,51006 |
| 3 | 0,45378 | 0,00083 | 0,54539 | 0,00000 | 0,00000 | 0,54539 |
| 1 | 0,39357 | 0,32557 | 0,28084 | 0,00001 | 0,00000 | 0,39357 |
| 2 | 0,40879 | 0,49697 | 0,00023 | 0,09401 | 0,00000 | 0,49697 |
| 1 | 0,52124 | 0,40293 | 0,07582 | 0,00000 | 0,00000 | 0,52124 |
| 1 | 0,56073 | 0,42850 | 0,00824 | 0,00253 | 0,00000 | 0,56073 |
| 4 | 0,00041 | 0,42592 | 0,00057 | 0,57309 | 0,00001 | 0,57309 |
| 3 | 0,17786 | 0,34691 | 0,47403 | 0,00121 | 0,00000 | 0,47403 |
| 1 | 0,57919 | 0,00000 | 0,42081 | 0,00000 | 0,00000 | 0,57919 |
| 2 | 0,40961 | 0,57461 | 0,01578 | 0,00000 | 0,00000 | 0,57461 |
| 4 | 0,00000 | 0,00000 | 0,35398 | 0,50550 | 0,14052 | 0,50550 |
| 3 | 0,38973 | 0,01157 | 0,59867 | 0,00002 | 0,00001 | 0,59867 |
| 3 | 0,30699 | 0,21363 | 0,47937 | 0,00000 | 0,00000 | 0,47937 |
| 3 | 0,31189 | 0,19846 | 0,48957 | 0,00008 | 0,00000 | 0,48957 |
| 2 | 0,00003 | 0,61848 | 0,00000 | 0,38149 | 0,00000 | 0,61848 |
| 2 | 0,20543 | 0,49248 | 0,30206 | 0,00002 | 0,00000 | 0,49248 |
| 3 | 0,37354 | 0,01736 | 0,60911 | 0,00000 | 0,00000 | 0,60911 |
| 2 | 0,34521 | 0,56844 | 0,08606 | 0,00029 | 0,00000 | 0,56844 |
| 3 | 0,37517 | 0,00002 | 0,62481 | 0,00000 | 0,00000 | 0,62481 |
| 3 | 0,00005 | 0,00008 | 0,61969 | 0,37103 | 0,00916 | 0,61969 |
| 3 | 0,35451 | 0,04440 | 0,60108 | 0,00000 | 0,00000 | 0,60108 |
| 3 | 0,00014 | 0,00001 | 0,62877 | 0,37083 | 0,00026 | 0,62877 |
| 1 | 0,59840 | 0,05099 | 0,35061 | 0,00000 | 0,00000 | 0,59840 |
| 3 | 0,00000 | 0,00000 | 0,58030 | 0,08643 | 0,33326 | 0,58030 |
| 3 | 0,26241 | 0,13944 | 0,46650 | 0,13164 | 0,00000 | 0,46650 |
| 3 | 0,10196 | 0,31893 | 0,57911 | 0,00000 | 0,00000 | 0,57911 |
