# Supplementary material for: Insomnia Subtypes in Clinical Population According to the Insomnia Type Questionnaire (ITQ): A Multi‐Centre Study in Spanish Sleep Clinics
Source: J Sleep Res. 2025 Jun 25;35(1):e70116. doi: 10.1111/jsr.70116 (PMC12856099; doi:10.1111/jsr.70116)
Supplement: Supplementary file 2 — Table S2. ITQ values of ‘pure’ patients (difference between the two highest certainty values, higher than 50%). [file JSR-35-e70116-s001.docx]

S2 table. ITQ values of “pure” patients (difference between the two highest certainty values, higher than 50%)

| Subtype | Subtype_1 | Subtype_2 | Subtype_3 | Subtype_4 | Subtype_5 | certainty |
| --- | --- | --- | --- | --- | --- | --- |
| 3 | 0,269680670 | 0,008211942 | 0,546806052 | 0,175301310 | 0,000000026 | 0,546806052 |
| 3 | 0,279760322 | 0,148469276 | 0,571770401 | 0,000000001 | 0,000000000 | 0,571770401 |
| 3 | 0,041696207 | 0,309531306 | 0,648772484 | 0,000000000 | 0,000000003 | 0,648772484 |
| 2 | 0,319746657 | 0,679999561 | 0,000253782 | 0,000000000 | 0,000000000 | 0,679999561 |
| 3 | 0,310707930 | 0,003160603 | 0,686036736 | 0,000093367 | 0,000001365 | 0,686036736 |
| 4 | 0,080637558 | 0,200086117 | 0,219301795 | 0,499673030 | 0,000301500 | 0,499673030 |
| 3 | 0,288852885 | 0,000074489 | 0,675373991 | 0,035695882 | 0,000002753 | 0,675373991 |
| 3 | 0,265901653 | 0,101265452 | 0,631102990 | 0,001729870 | 0,000000036 | 0,631102990 |
| 3 | 0,000754583 | 0,000013482 | 0,711950129 | 0,287223726 | 0,000058080 | 0,711950129 |
| 3 | 0,005277283 | 0,242982227 | 0,623132748 | 0,128592705 | 0,000015036 | 0,623132748 |
| 3 | 0,171024260 | 0,229368839 | 0,599606888 | 0,000000000 | 0,000000012 | 0,599606888 |
| 3 | 0,093401366 | 0,250229712 | 0,656368922 | 0,000000000 | 0,000000000 | 0,656368922 |
| 3 | 0,258229581 | 0,024689391 | 0,716420999 | 0,000660024 | 0,000000005 | 0,716420999 |
| 4 | 0,000026661 | 0,001820586 | 0,253823260 | 0,743894294 | 0,000435199 | 0,743894294 |
| 1 | 0,764014406 | 0,000000000 | 0,235985594 | 0,000000000 | 0,000000000 | 0,764014406 |
| 3 | 0,211197119 | 0,095645908 | 0,692843937 | 0,000312091 | 0,000000944 | 0,692843937 |
| 2 | 0,075308593 | 0,708903619 | 0,215783776 | 0,000000002 | 0,000004010 | 0,708903619 |
| 2 | 0,218128089 | 0,741590175 | 0,040281736 | 0,000000000 | 0,000000001 | 0,741590175 |
| 1 | 0,692849883 | 0,104083089 | 0,198001754 | 0,005065270 | 0,000000004 | 0,692849883 |
| 3 | 0,217946083 | 0,001358986 | 0,780694932 | 0,000000000 | 0,000000000 | 0,780694932 |
| 3 | 0,214593537 | 0,000000332 | 0,785406130 | 0,000000000 | 0,000000000 | 0,785406130 |
| 3 | 0,000561628 | 0,017229710 | 0,766410667 | 0,009128323 | 0,206669671 | 0,766410667 |
| 2 | 0,003349802 | 0,781257315 | 0,018243316 | 0,197149306 | 0,000000261 | 0,781257315 |
| 5 | 0,000003210 | 0,000327524 | 0,190868435 | 0,024947500 | 0,783853331 | 0,783853331 |
| 3 | 0,193903439 | 0,000231732 | 0,805864825 | 0,000000004 | 0,000000000 | 0,805864825 |
| 1 | 0,808295709 | 0,000000000 | 0,191704291 | 0,000000000 | 0,000000000 | 0,808295709 |
| 1 | 0,796051689 | 0,019878441 | 0,184068708 | 0,000001163 | 0,000000000 | 0,796051689 |
| 1 | 0,820064439 | 0,179933460 | 0,000002101 | 0,000000000 | 0,000000000 | 0,820064439 |
| 2 | 0,080631167 | 0,756742398 | 0,162625672 | 0,000000755 | 0,000000008 | 0,756742398 |
| 2 | 0,014623763 | 0,801545063 | 0,014380607 | 0,169359627 | 0,000090940 | 0,801545063 |
| 2 | 0,156578276 | 0,763445196 | 0,079976528 | 0,000000000 | 0,000000000 | 0,763445196 |
| 4 | 0,000216864 | 0,000350289 | 0,164358937 | 0,835073859 | 0,000000051 | 0,835073859 |
| 1 | 0,831031746 | 0,160939564 | 0,008028656 | 0,000000033 | 0,000000001 | 0,831031746 |
| 4 | 0,152689181 | 0,012724511 | 0,002099306 | 0,832486944 | 0,000000058 | 0,832486944 |
| 3 | 0,152024406 | 0,000000484 | 0,847975109 | 0,000000000 | 0,000000000 | 0,847975109 |
| 3 | 0,151626316 | 0,000858319 | 0,847513161 | 0,000000295 | 0,000001909 | 0,847513161 |
| 3 | 0,151560878 | 0,000000000 | 0,848439122 | 0,000000000 | 0,000000000 | 0,848439122 |
| 3 | 0,148118279 | 0,000746576 | 0,851133926 | 0,000001219 | 0,000000000 | 0,851133926 |
| 3 | 0,139472287 | 0,000000063 | 0,860527650 | 0,000000000 | 0,000000000 | 0,860527650 |
| 3 | 0,000131608 | 0,007158672 | 0,857240656 | 0,134542962 | 0,000926101 | 0,857240656 |
| 1 | 0,874307446 | 0,125663517 | 0,000027331 | 0,000001706 | 0,000000000 | 0,874307446 |
| 2 | 0,000759282 | 0,883761714 | 0,000007788 | 0,115471197 | 0,000000019 | 0,883761714 |
| 1 | 0,883934277 | 0,114969475 | 0,001096247 | 0,000000000 | 0,000000000 | 0,883934277 |
| 2 | 0,000007461 | 0,888849595 | 0,000393558 | 0,110738286 | 0,000011100 | 0,888849595 |
| 3 | 0,050212707 | 0,104205255 | 0,845582002 | 0,000000001 | 0,000000035 | 0,845582002 |
| 3 | 0,002524688 | 0,000427677 | 0,888368572 | 0,108679061 | 0,000000002 | 0,888368572 |
| 2 | 0,104172733 | 0,893533744 | 0,000330246 | 0,001963265 | 0,000000012 | 0,893533744 |
| 3 | 0,094829520 | 0,005783834 | 0,832850996 | 0,066535626 | 0,000000024 | 0,832850996 |
| 1 | 0,866403038 | 0,036495978 | 0,097100984 | 0,000000000 | 0,000000000 | 0,866403038 |
| 3 | 0,100231042 | 0,000351009 | 0,899417949 | 0,000000000 | 0,000000000 | 0,899417949 |
| 2 | 0,006256501 | 0,896169147 | 0,096712153 | 0,000050417 | 0,000811781 | 0,896169147 |
| 3 | 0,003958729 | 0,088316669 | 0,864902136 | 0,026152301 | 0,016670166 | 0,864902136 |
| 1 | 0,867996235 | 0,047850504 | 0,084153262 | 0,000000000 | 0,000000000 | 0,867996235 |
| 3 | 0,087429135 | 0,000003672 | 0,912567193 | 0,000000000 | 0,000000000 | 0,912567193 |
| 3 | 0,087175503 | 0,001321070 | 0,911503427 | 0,000000000 | 0,000000000 | 0,911503427 |
| 3 | 0,078933468 | 0,030675599 | 0,890388068 | 0,000000224 | 0,000002640 | 0,890388068 |
| 1 | 0,913395673 | 0,005679931 | 0,080924396 | 0,000000000 | 0,000000000 | 0,913395673 |
| 1 | 0,922413352 | 0,076927332 | 0,000659316 | 0,000000000 | 0,000000000 | 0,922413352 |
| 1 | 0,925972626 | 0,001427648 | 0,072599726 | 0,000000000 | 0,000000000 | 0,925972626 |
| 1 | 0,912597381 | 0,019625375 | 0,067777244 | 0,000000000 | 0,000000000 | 0,912597381 |
| 3 | 0,062951016 | 0,026756050 | 0,910292914 | 0,000000019 | 0,000000000 | 0,910292914 |
| 3 | 0,060166737 | 0,000000247 | 0,939833016 | 0,000000000 | 0,000000000 | 0,939833016 |
| 3 | 0,000024323 | 0,000305230 | 0,939529196 | 0,060110773 | 0,000030478 | 0,939529196 |
| 3 | 0,058113530 | 0,000012536 | 0,941873933 | 0,000000000 | 0,000000000 | 0,941873933 |
| 3 | 0,048663291 | 0,005491401 | 0,892671686 | 0,053165590 | 0,000008032 | 0,892671686 |
| 3 | 0,055249886 | 0,000020959 | 0,944729155 | 0,000000000 | 0,000000000 | 0,944729155 |
| 1 | 0,945390844 | 0,000276140 | 0,054333016 | 0,000000000 | 0,000000000 | 0,945390844 |
| 1 | 0,913743652 | 0,036453577 | 0,049802771 | 0,000000000 | 0,000000000 | 0,913743652 |
| 3 | 0,000224816 | 0,000103260 | 0,948437000 | 0,002966156 | 0,048268767 | 0,948437000 |
| 3 | 0,003456551 | 0,047365094 | 0,946661264 | 0,002478275 | 0,000038816 | 0,946661264 |
| 2 | 0,046040836 | 0,925610082 | 0,028349082 | 0,000000000 | 0,000000000 | 0,925610082 |
| 4 | 0,000000317 | 0,045706111 | 0,020272648 | 0,931913836 | 0,002107089 | 0,931913836 |
| 1 | 0,912947700 | 0,043193917 | 0,043858383 | 0,000000000 | 0,000000000 | 0,912947700 |
| 1 | 0,953956941 | 0,000729416 | 0,045313641 | 0,000000002 | 0,000000000 | 0,953956941 |
| 2 | 0,003185214 | 0,951712565 | 0,002844262 | 0,042257960 | 0,000000000 | 0,951712565 |
| 3 | 0,017469607 | 0,015681597 | 0,925829415 | 0,040882686 | 0,000136696 | 0,925829415 |
| 3 | 0,012522301 | 0,039341746 | 0,948035128 | 0,000004143 | 0,000096682 | 0,948035128 |
| 3 | 0,037341531 | 0,000001157 | 0,962657049 | 0,000000263 | 0,000000000 | 0,962657049 |
| 1 | 0,933007472 | 0,031570576 | 0,035375715 | 0,000046237 | 0,000000000 | 0,933007472 |
| 3 | 0,034557246 | 0,000169133 | 0,965271870 | 0,000001735 | 0,000000016 | 0,965271870 |
| 2 | 0,034473427 | 0,964585630 | 0,000940943 | 0,000000000 | 0,000000000 | 0,964585630 |
| 3 | 0,033163427 | 0,000000062 | 0,966836507 | 0,000000004 | 0,000000000 | 0,966836507 |
| 3 | 0,033022118 | 0,000117605 | 0,964360430 | 0,002499847 | 0,000000000 | 0,964360430 |
| 1 | 0,967538822 | 0,000005474 | 0,032455704 | 0,000000000 | 0,000000000 | 0,967538822 |
| 3 | 0,000635907 | 0,031897165 | 0,966440320 | 0,000462050 | 0,000564557 | 0,966440320 |
| 1 | 0,969736997 | 0,000011724 | 0,030251278 | 0,000000000 | 0,000000000 | 0,969736997 |
| 2 | 0,000056665 | 0,964730756 | 0,005915115 | 0,029190230 | 0,000107234 | 0,964730756 |
| 4 | 0,000003532 | 0,026414887 | 0,002160481 | 0,971347950 | 0,000073151 | 0,971347950 |
| 2 | 0,025544681 | 0,955327790 | 0,001775757 | 0,017350937 | 0,000000834 | 0,955327790 |
| 1 | 0,974207253 | 0,000000822 | 0,025791925 | 0,000000000 | 0,000000000 | 0,974207253 |
| 3 | 0,024872691 | 0,022357059 | 0,952770157 | 0,000000051 | 0,000000042 | 0,952770157 |
| 1 | 0,972142301 | 0,003147413 | 0,024710286 | 0,000000000 | 0,000000000 | 0,972142301 |
| 2 | 0,000977620 | 0,974577378 | 0,000002978 | 0,024441721 | 0,000000303 | 0,974577378 |
| 1 | 0,955026154 | 0,021058528 | 0,023915292 | 0,000000025 | 0,000000000 | 0,955026154 |
| 4 | 0,002555293 | 0,023204027 | 0,005399625 | 0,968833190 | 0,000007866 | 0,968833190 |
| 1 | 0,974248047 | 0,022986754 | 0,002765199 | 0,000000000 | 0,000000000 | 0,974248047 |
| 2 | 0,000693103 | 0,970936283 | 0,006068857 | 0,022301700 | 0,000000058 | 0,970936283 |
| 3 | 0,021916531 | 0,014396254 | 0,963687215 | 0,000000000 | 0,000000000 | 0,963687215 |
| 3 | 0,000122669 | 0,000025338 | 0,978953030 | 0,020896089 | 0,000002874 | 0,978953030 |
| 3 | 0,020677045 | 0,000000000 | 0,979322955 | 0,000000000 | 0,000000000 | 0,979322955 |
| 1 | 0,980636304 | 0,000000005 | 0,019363691 | 0,000000000 | 0,000000000 | 0,980636304 |
| 4 | 0,000004028 | 0,019157445 | 0,007367946 | 0,973386048 | 0,000084533 | 0,973386048 |
| 4 | 0,000097159 | 0,010376424 | 0,016147719 | 0,954867213 | 0,018511485 | 0,954867213 |
| 2 | 0,000356780 | 0,980053576 | 0,018454671 | 0,001095386 | 0,000039587 | 0,980053576 |
| 1 | 0,981945749 | 0,017993823 | 0,000060388 | 0,000000040 | 0,000000000 | 0,981945749 |
| 3 | 0,016060982 | 0,000931773 | 0,983007241 | 0,000000000 | 0,000000004 | 0,983007241 |
| 3 | 0,000003339 | 0,000000475 | 0,984022798 | 0,015930570 | 0,000042818 | 0,984022798 |
| 1 | 0,984172754 | 0,000012868 | 0,015814378 | 0,000000000 | 0,000000000 | 0,984172754 |
| 4 | 0,000000007 | 0,015744233 | 0,000054801 | 0,984056416 | 0,000144543 | 0,984056416 |
| 3 | 0,015328925 | 0,000000369 | 0,984670705 | 0,000000000 | 0,000000000 | 0,984670705 |
| 3 | 0,015045713 | 0,006665277 | 0,978248993 | 0,000040009 | 0,000000008 | 0,978248993 |
| 2 | 0,014559990 | 0,980545785 | 0,003875176 | 0,001017826 | 0,000001223 | 0,980545785 |
| 1 | 0,974065166 | 0,012046507 | 0,013888327 | 0,000000000 | 0,000000000 | 0,974065166 |
| 1 | 0,985976988 | 0,000000009 | 0,014023003 | 0,000000000 | 0,000000000 | 0,985976988 |
| 1 | 0,986506977 | 0,000000099 | 0,013492924 | 0,000000000 | 0,000000000 | 0,986506977 |
| 3 | 0,013381199 | 0,000084413 | 0,986534388 | 0,000000000 | 0,000000000 | 0,986534388 |
| 3 | 0,012822768 | 0,000053695 | 0,987123537 | 0,000000000 | 0,000000000 | 0,987123537 |
| 1 | 0,987216182 | 0,012495907 | 0,000287911 | 0,000000000 | 0,000000000 | 0,987216182 |
| 3 | 0,000336916 | 0,000005846 | 0,987612073 | 0,011943628 | 0,000101537 | 0,987612073 |
| 1 | 0,986409726 | 0,011919110 | 0,001671165 | 0,000000000 | 0,000000000 | 0,986409726 |
| 3 | 0,010795134 | 0,000015039 | 0,989189530 | 0,000000000 | 0,000000296 | 0,989189530 |
| 3 | 0,010308188 | 0,000000353 | 0,989691457 | 0,000000001 | 0,000000001 | 0,989691457 |
| 1 | 0,988058918 | 0,001709212 | 0,010137298 | 0,000094572 | 0,000000000 | 0,988058918 |
| 3 | 0,009437496 | 0,000000957 | 0,990561544 | 0,000000002 | 0,000000000 | 0,990561544 |
| 1 | 0,990625451 | 0,009374547 | 0,000000002 | 0,000000000 | 0,000000000 | 0,990625451 |
| 3 | 0,008905777 | 0,002887196 | 0,988203739 | 0,000001972 | 0,000001316 | 0,988203739 |
| 3 | 0,008780004 | 0,000001933 | 0,991217326 | 0,000000737 | 0,000000000 | 0,991217326 |
| 2 | 0,000102047 | 0,990283858 | 0,008620755 | 0,000992796 | 0,000000544 | 0,990283858 |
| 2 | 0,008546252 | 0,982733355 | 0,008500247 | 0,000220057 | 0,000000089 | 0,982733355 |
| 1 | 0,991458024 | 0,000122452 | 0,000380006 | 0,008039518 | 0,000000000 | 0,991458024 |
| 1 | 0,991045999 | 0,000306638 | 0,001168471 | 0,007478892 | 0,000000000 | 0,991045999 |
| 3 | 0,006461438 | 0,000000000 | 0,993538562 | 0,000000000 | 0,000000000 | 0,993538562 |
| 3 | 0,000198843 | 0,000036939 | 0,991140929 | 0,002215499 | 0,006407790 | 0,991140929 |
| 1 | 0,993648833 | 0,006278554 | 0,000072613 | 0,000000000 | 0,000000000 | 0,993648833 |
| 3 | 0,006229993 | 0,000574309 | 0,993129731 | 0,000065898 | 0,000000070 | 0,993129731 |
| 3 | 0,006124055 | 0,000006122 | 0,993869823 | 0,000000000 | 0,000000000 | 0,993869823 |
| 1 | 0,994571335 | 0,000026391 | 0,005402273 | 0,000000000 | 0,000000000 | 0,994571335 |
| 3 | 0,005309544 | 0,000017075 | 0,994250949 | 0,000422419 | 0,000000013 | 0,994250949 |
| 1 | 0,994908029 | 0,000000002 | 0,005091969 | 0,000000000 | 0,000000000 | 0,994908029 |
| 3 | 0,004445547 | 0,000101254 | 0,995358037 | 0,000095162 | 0,000000000 | 0,995358037 |
| 3 | 0,004225363 | 0,000355869 | 0,995418767 | 0,000000001 | 0,000000000 | 0,995418767 |
| 1 | 0,995413186 | 0,000392495 | 0,004194319 | 0,000000000 | 0,000000000 | 0,995413186 |
| 1 | 0,994290905 | 0,001626911 | 0,004082184 | 0,000000000 | 0,000000000 | 0,994290905 |
| 1 | 0,996039027 | 0,000007414 | 0,003953559 | 0,000000000 | 0,000000000 | 0,996039027 |
| 3 | 0,000000704 | 0,000001950 | 0,994201384 | 0,001963923 | 0,003832039 | 0,994201384 |
| 1 | 0,994726931 | 0,001476485 | 0,003796584 | 0,000000000 | 0,000000000 | 0,994726931 |
| 1 | 0,995500062 | 0,003653374 | 0,000846564 | 0,000000000 | 0,000000000 | 0,995500062 |
| 3 | 0,000966520 | 0,003644266 | 0,994420746 | 0,000964275 | 0,000004192 | 0,994420746 |
| 1 | 0,996801210 | 0,000271990 | 0,002926799 | 0,000000000 | 0,000000000 | 0,996801210 |
| 3 | 0,002403605 | 0,000043261 | 0,997196488 | 0,000356634 | 0,000000013 | 0,997196488 |
| 3 | 0,000029835 | 0,000034779 | 0,997661004 | 0,000005651 | 0,002268731 | 0,997661004 |
| 2 | 0,001980541 | 0,995505466 | 0,000291272 | 0,002222687 | 0,000000035 | 0,995505466 |
| 1 | 0,997554623 | 0,000328402 | 0,002116975 | 0,000000000 | 0,000000000 | 0,997554623 |
| 3 | 0,000192031 | 0,000013444 | 0,997861715 | 0,001910199 | 0,000022611 | 0,997861715 |
| 3 | 0,001837067 | 0,000232347 | 0,997930586 | 0,000000000 | 0,000000000 | 0,997930586 |
| 3 | 0,001559573 | 0,000307399 | 0,997072691 | 0,001037879 | 0,000022458 | 0,997072691 |
| 1 | 0,998490617 | 0,001500859 | 0,000008524 | 0,000000000 | 0,000000000 | 0,998490617 |
| 3 | 0,000000769 | 0,000000002 | 0,998380152 | 0,001457258 | 0,000161820 | 0,998380152 |
| 3 | 0,001451299 | 0,000013120 | 0,998535581 | 0,000000000 | 0,000000000 | 0,998535581 |
| 3 | 0,001365112 | 0,000130358 | 0,997598277 | 0,000186919 | 0,000719334 | 0,997598277 |
| 1 | 0,997696669 | 0,001192819 | 0,000036377 | 0,001074135 | 0,000000000 | 0,997696669 |
| 3 | 0,001117290 | 0,000102742 | 0,997311754 | 0,001065553 | 0,000402661 | 0,997311754 |
| 1 | 0,998124018 | 0,000760852 | 0,001107075 | 0,000008055 | 0,000000000 | 0,998124018 |
| 1 | 0,998959702 | 0,000000125 | 0,001040173 | 0,000000000 | 0,000000000 | 0,998959702 |
| 3 | 0,000107841 | 0,000001946 | 0,998945875 | 0,000907491 | 0,000036847 | 0,998945875 |
| 4 | 0,000021726 | 0,000882771 | 0,000502220 | 0,998589106 | 0,000004177 | 0,998589106 |
| 3 | 0,000779287 | 0,000002386 | 0,999200545 | 0,000017526 | 0,000000257 | 0,999200545 |
| 3 | 0,000605656 | 0,000004296 | 0,998748388 | 0,000640553 | 0,000001107 | 0,998748388 |
| 1 | 0,999479821 | 0,000000000 | 0,000520179 | 0,000000000 | 0,000000000 | 0,999479821 |
| 3 | 0,000257142 | 0,000343148 | 0,998522903 | 0,000500968 | 0,000375839 | 0,998522903 |
| 3 | 0,000491945 | 0,000000005 | 0,999508050 | 0,000000000 | 0,000000000 | 0,999508050 |
| 1 | 0,999566646 | 0,000000002 | 0,000433352 | 0,000000000 | 0,000000000 | 0,999566646 |
| 4 | 0,000000317 | 0,000170084 | 0,000155588 | 0,999259833 | 0,000414178 | 0,999259833 |
| 1 | 0,999564296 | 0,000022516 | 0,000413187 | 0,000000000 | 0,000000000 | 0,999564296 |
| 3 | 0,000000730 | 0,000010527 | 0,999477078 | 0,000410658 | 0,000101007 | 0,999477078 |
| 3 | 0,000390599 | 0,000000018 | 0,999215502 | 0,000393882 | 0,000000000 | 0,999215502 |
| 1 | 0,999613621 | 0,000372217 | 0,000014162 | 0,000000000 | 0,000000000 | 0,999613621 |
| 3 | 0,000000159 | 0,000000001 | 0,999615890 | 0,000342046 | 0,000041904 | 0,999615890 |
| 3 | 0,000283111 | 0,000236053 | 0,999436261 | 0,000028150 | 0,000016425 | 0,999436261 |
| 3 | 0,000110814 | 0,000278541 | 0,999602354 | 0,000008052 | 0,000000239 | 0,999602354 |
| 2 | 0,000015578 | 0,999665895 | 0,000273336 | 0,000000362 | 0,000044829 | 0,999665895 |
| 3 | 0,000022024 | 0,000257701 | 0,999674817 | 0,000020333 | 0,000025123 | 0,999674817 |
| 3 | 0,000147821 | 0,000218693 | 0,999599838 | 0,000033161 | 0,000000487 | 0,999599838 |
| 3 | 0,000001272 | 0,000001329 | 0,999774013 | 0,000009891 | 0,000213495 | 0,999774013 |
| 3 | 0,000201340 | 0,000004661 | 0,999754234 | 0,000000496 | 0,000039269 | 0,999754234 |
| 3 | 0,000001709 | 0,000000001 | 0,999806008 | 0,000191673 | 0,000000610 | 0,999806008 |
| 1 | 0,999811496 | 0,000177626 | 0,000010879 | 0,000000000 | 0,000000000 | 0,999811496 |
| 1 | 0,999726802 | 0,000102690 | 0,000170507 | 0,000000000 | 0,000000000 | 0,999726802 |
| 1 | 0,999848374 | 0,000121884 | 0,000029742 | 0,000000000 | 0,000000000 | 0,999848374 |
| 2 | 0,000118710 | 0,999870938 | 0,000007260 | 0,000003092 | 0,000000000 | 0,999870938 |
| 1 | 0,999908019 | 0,000000000 | 0,000091981 | 0,000000000 | 0,000000000 | 0,999908019 |
| 1 | 0,999893663 | 0,000018173 | 0,000088164 | 0,000000000 | 0,000000000 | 0,999893663 |
| 3 | 0,000088067 | 0,000000000 | 0,999911933 | 0,000000000 | 0,000000000 | 0,999911933 |
| 1 | 0,999895648 | 0,000067091 | 0,000037261 | 0,000000000 | 0,000000000 | 0,999895648 |
| 3 | 0,000064555 | 0,000000030 | 0,999934504 | 0,000000907 | 0,000000003 | 0,999934504 |
| 3 | 0,000063477 | 0,000050324 | 0,999847551 | 0,000008875 | 0,000029773 | 0,999847551 |
| 3 | 0,000001484 | 0,000000001 | 0,999943333 | 0,000053444 | 0,000001738 | 0,999943333 |
| 1 | 0,999932490 | 0,000027390 | 0,000040120 | 0,000000000 | 0,000000000 | 0,999932490 |
| 3 | 0,000003992 | 0,000004099 | 0,999973684 | 0,000000039 | 0,000018186 | 0,999973684 |
| 1 | 0,999981842 | 0,000000000 | 0,000018158 | 0,000000000 | 0,000000000 | 0,999981842 |
| 1 | 0,999984456 | 0,000015413 | 0,000000131 | 0,000000000 | 0,000000000 | 0,999984456 |
| 1 | 0,999980281 | 0,000015405 | 0,000004315 | 0,000000000 | 0,000000000 | 0,999980281 |
| 1 | 0,999988771 | 0,000000000 | 0,000011229 | 0,000000000 | 0,000000000 | 0,999988771 |
| 1 | 0,999987031 | 0,000009960 | 0,000003009 | 0,000000000 | 0,000000000 | 0,999987031 |
| 3 | 0,000009815 | 0,000000000 | 0,999990185 | 0,000000000 | 0,000000000 | 0,999990185 |
| 3 | 0,000002210 | 0,000003741 | 0,999986649 | 0,000000024 | 0,000007377 | 0,999986649 |
| 1 | 0,999994225 | 0,000000007 | 0,000005768 | 0,000000000 | 0,000000000 | 0,999994225 |
| 3 | 0,000000011 | 0,000000000 | 0,999995369 | 0,000004541 | 0,000000078 | 0,999995369 |
| 1 | 0,999995714 | 0,000000007 | 0,000004279 | 0,000000000 | 0,000000000 | 0,999995714 |
| 1 | 0,999995723 | 0,000000000 | 0,000004276 | 0,000000000 | 0,000000000 | 0,999995723 |
| 4 | 0,000004177 | 0,000000363 | 0,000000019 | 0,999995441 | 0,000000000 | 0,999995441 |
| 1 | 0,999995671 | 0,000000263 | 0,000004066 | 0,000000000 | 0,000000000 | 0,999995671 |
| 1 | 0,999994776 | 0,000003860 | 0,000001364 | 0,000000000 | 0,000000000 | 0,999994776 |
| 1 | 0,999996732 | 0,000000000 | 0,000003268 | 0,000000000 | 0,000000000 | 0,999996732 |
| 1 | 0,999997963 | 0,000001386 | 0,000000651 | 0,000000000 | 0,000000000 | 0,999997963 |
| 3 | 0,000000330 | 0,000000000 | 0,999998417 | 0,000001243 | 0,000000010 | 0,999998417 |
| 3 | 0,000000536 | 0,000000357 | 0,999999003 | 0,000000007 | 0,000000097 | 0,999999003 |
| 1 | 0,999999382 | 0,000000276 | 0,000000342 | 0,000000000 | 0,000000000 | 0,999999382 |
| 3 | 0,000000279 | 0,000000000 | 0,999999718 | 0,000000000 | 0,000000003 | 0,999999718 |
| 1 | 0,999999850 | 0,000000015 | 0,000000135 | 0,000000000 | 0,000000000 | 0,999999850 |
| 1 | 0,999999812 | 0,000000055 | 0,000000134 | 0,000000000 | 0,000000000 | 0,999999812 |
| 1 | 0,999999979 | 0,000000020 | 0,000000000 | 0,000000000 | 0,000000000 | 0,999999979 |
| 1 | 0,999999975 | 0,000000018 | 0,000000007 | 0,000000000 | 0,000000000 | 0,999999975 |
| 1 | 0,999999996 | 0,000000001 | 0,000000003 | 0,000000000 | 0,000000000 | 0,999999996 |
| 1 | 0,999999997 | 0,000000002 | 0,000000001 | 0,000000000 | 0,000000000 | 0,999999997 |
| 1 | 0,999999999 | 0,000000001 | 0,000000000 | 0,000000000 | 0,000000000 | 0,999999999 |
